# Moderate Reverberation Does Not Increase Subjective Fatigue, Subjective Listening Effort, or Behavioral Listening Effort in School-Aged Children

**DOI:** 10.3389/fpsyg.2019.01749

**Published:** 2019-08-02

**Authors:** Erin M. Picou, Brianna Bean, Steven C. Marcrum, Todd A. Ricketts, Benjamin W. Y. Hornsby

**Affiliations:** ^1^Hearing and Affect Perception Interest Laboratory, Department of Hearing and Speech Sciences, Vanderbilt University Medical Center, Nashville, TN, United States; ^2^Dan Maddox Hearing Aid Research Laboratory, Department of Hearing and Speech Sciences, Vanderbilt University Medical Center, Nashville, TN, United States; ^3^Department of Otolaryngology, University Hospital Regensburg, Regensburg, Germany; ^4^Hearing and Communication Laboratory, Department of Hearing and Speech Sciences, Vanderbilt University Medical Center, Nashville, TN, United States

**Keywords:** children, classrooms, background noise, listening effort, subjective ratings, reverberation, speech recognition

## Abstract

Background noise and reverberation levels in typical classrooms have negative effects on speech recognition, but their effects on listening effort and fatigue are less well understood. Based on the Framework for Understanding Effortful Listening, noise and reverberation would be expected to increase both listening effort and fatigue. However, previous investigations of the effects of reverberation for adults have resulted in mixed findings. Some discrepancies in the literature might be accounted for by methodological differences; behavioral and subjective indices of listening effort do not often align in adults. The effects of sustained listening on self-reported fatigue in school-aged children are also not well understood. The purposes of this project were to (1) evaluate the effects of noise and reverberation on listening effort in school-aged children using behavioral and subjective measures, (2) compare subjective and behavioral indices of listening effort, and (3) evaluate the effects of reverberation on self-reported fatigue. Twenty typically developing children (10–17 years old) participated. Participants completed dual-task testing in two rooms that varied in terms of reverberation, an audiometric sound booth and a moderately reverberant room. In each room, testing was completed in quiet and in two levels of background noise. Participants provided subjective ratings of listening effort after completing the dual-task in each listening condition. Subjective ratings of fatigue were completed before and after testing in each level of reverberation. Results revealed background noise, not reverberation, increased behavioral and subjective listening effort. Subjective ratings of perceived performance, ease of listening, and desire to control the listening situation revealed a similar pattern of results as word recognition performance, making them poor candidates for providing an indication of behavioral listening effort. However, ratings of time perception were moderately correlated with behavioral listening effort. Finally, sustained listening for approximately 25 min increased self-reported fatigue, although changes in fatigue were comparable in low and moderately reverberant environments. In total, these data offer no evidence that a moderate level of reverberation increases listening effort or fatigue, but the data do support the reduction of background noise in classrooms.

## Introduction

For school-aged children, listening in classrooms can be challenging. Typical classroom environments are acoustically disadvantaged with signal-to-noise ratios (SNRs) ranging from -6 to +13 dB (Pearsons et al., 1977; Bradley and Sato, 2008; Sato and Bradley, 2008), whereas ideal SNRs for classrooms are considerably more favorable (e.g., +15 to +30; Berg, 1993; Bistafa and Bradley, 2000; Crandell and Smaldino, 2000). In addition, typical classrooms are likely to be more reverberant than is recommended, with measured classroom reverberation times of 600 ms (Crandell and Smaldino, 1994; Crukley et al., 2011) to 1200 ms (Crandell and Smaldino, 1994), whereas reverberation times of 400 to 500 ms or less are recommended (Finitzo-Hieber and Tillman, 1978; Bistafa and Bradley, 2000).

The perceptual consequences of listening in acoustically disadvantaged environments include not only reduced speech recognition, but also increased listening effort (e.g., Prodi et al., 2010). “Listening effort” is defined as the “deliberate allocation of resources to overcome obstacles in goal pursuit” when listening (Pichora-Fuller et al., 2016, pg. 11S). Given the important, negative consequences of sustained increases in listening effort, such as communicative disengagement (Hétu et al., 1988), reduced vocational involvement (Kramer et al., 2006), and mental fatigue (Hornsby, 2013), it is important to understand the factors that affect listening effort.

The Ease of Language Understanding (ELU) model (Rönnberg et al., 2008, 2013) provides a framework for understanding listening effort. Briefly, the model suggests that a listener compares language inputs to long-term memory stores. Understanding is easy or effortless if the language input matches a long-term memory store. Conversely, if a match is not immediate, cognitive resources must be deployed to facilitate a match. The Framework for Understanding Effortful Listening (FUEL; Pichora-Fuller et al., 2016), based on the model of limited attention proposed by Kahneman (1973), extends the ELU model by including elements of executive function that control a resource allocation policy. Specifically, cognitive resources can be allocated automatically (e.g., in response to sudden stimuli), intentionally (e.g., with explicit instruction), or evaluatively (e.g., to attain a goal). Assuming the allocation is consistent across listening conditions, both the ELU and FUEL frameworks suggest that factors interfere with the input-memory match, such as background noise and reverberation, would increase listening effort.

Consistent with the hypothesis that background noise interferes with an input-memory match and thus requires deployment of cognitive resources, investigators have repeatedly demonstrated increased listening effort in adults with the addition, or increased level, of background noise. Effects of background noise on listening effort have been demonstrated with memory paradigms (Surprenant, 1999; Murphy et al., 2000; Picou et al., 2011), physiologic measures (Zekveld et al., 2010, 2011; Mackersie and Cones, 2011), and behavioral reaction-time measures (Sarampalis et al., 2009; Fraser et al., 2010; Picou et al., 2013).

In school-aged children, the results of studies into the effects of background noise on listening effort are less consistent. Using behavioral reaction-time tasks, some investigators have reported that SNR improvements (i.e., decreasing background noise levels) reduces listening effort (Prodi et al., 2010; Gustafson et al., 2014; Lewis et al., 2016; Hsu et al., 2017; McGarrigle et al., 2019); however, the finding is not universal (Hicks and Tharpe, 2002; Howard et al., 2010; McGarrigle et al., 2017, 2019). Some of the discrepancy between the published findings and ELU and FUEL predictions might be related to the sensitivity of the various listening effort paradigms. If a task is not motivating or is too distracting, changes in listening effort will be less evident (Choi et al., 2008), as might have been the case in earlier investigations of effort in school-aged children (e.g., McFadden and Pittman, 2008). When utilizing secondary tasks that are moderately challenging, investigators have found changes in behavioral effort with changes in SNR (Hsu et al., 2017; Picou et al., 2017a).

According to the ELU and FUEL frameworks, another transmission factor expected to increase listening effort, and relevant to contemporary classrooms, is room reverberation. Reverberation effects are generally described as either “early” or “late,” based on their time of arrival to a listener’s ear. Early reflections, or those that arrive within 0.05 s after direct signal presentation (Bradley, 1986; Bradley et al., 1999), are integrated with direct signal energy (Haas, 1972; Nábelek and Robinette, 1978). Late reflections, however, are not integrated with the direct signal energy and instead result in masking and temporal smearing of the original signal. As a result, late reflections reduce speech recognition performance, particularly in the middle part of the performance-intensity function (Nábělek and Pickett, 1974; Finitzo-Hieber and Tillman, 1978; Neuman et al., 2010; Wróblewski et al., 2012). Thus, one would also expect increased listening effort associated with reverberation.

However, the observed effects of reverberation on listening effort are unclear. For adults with normal hearing, several investigators have reported that increased levels of reverberation result in increased listening effort, as measured via subjective ratings with recorded stimuli (Sato et al., 2008, 2012; Rennies et al., 2014). However, other investigators using behavioral paradigms have failed to demonstrate increased listening effort with moderate increases in reverberation (Picou et al., 2016; Peng and Wang, 2019). For example, Picou et al. (2016) found that increasing reverberation (from <100 to 475 or to 834 ms), did not increase listening effort for adults with normal hearing. Explanations for the non-significant effects of reverberation remain elusive. It is possible reverberation affects listening effort only for some acoustic conditions, such as with multiple, moving talkers (Valente et al., 2012) or with longer reverberation times (e.g., T30 > 900 ms). It is also possible listening difficulties associated with listening to distortions are fundamentally different than the listening difficulties associated with noise masking, as suggested by Francis et al. (2016).

Importantly, there is scarce literature reporting on the effects of reverberation on listening effort for school-aged children. In terms of speech recognition, children are more vulnerable to the effects of reverberation than adults (Klatte et al., 2010b; Neuman et al., 2010; Valente et al., 2012; Wróblewski et al., 2012). In addition, evidence from real classrooms demonstrates negative effects of longer reverberation times (1000 compared to <500 ms) on students’ phonological processing, noise annoyance ratings, and teacher relationships (Klatte et al., 2010a). Thus, it is possible that reverberation could increase listening effort in school-aged children, despite non-significant behavioral findings in adults.

Alternatively, Amlani and Russo (2016) found that adding acoustic paneling to reduce reverberation in a classroom *increased* listening effort, as measured using a recall-based, dual-task paradigm in 8 to 9-year-old children with normal hearing. The authors attributed this negative effect to a combination of loss of early reflections and seat positions outside the critical distance. Combined with the findings in adults, the data from Amlani and Russo provide support for the competing hypothesis that reverberation will not increase listening effort in school-aged children.

Note that increases in reverberation resulted in increased listening effort in adults using subjective paradigms but not using behavioral paradigms. This discrepancy might be attributable to the different listening effort methodologies. Physiology (e.g., pupillometry) and behavioral (e.g., recall and response time) measures have been shown to be sensitive, indirect, indicators of listening effort (McGarrigle et al., 2014; Strand et al., 2018). While subjective ratings are assumed to provide a more direct estimate of an individual’s perceived listening effort, these ratings are often not associated with behavioral or physiologic measures (e.g., Feuerstein, 1992; Zekveld et al., 2010; Lemke and Besser, 2016; Picou et al., 2017b; Strand et al., 2018).

One explanation for the disparate findings is humans are not inherently disposed to accurately rate their listening effort; assigning a value to the “deliberate allocation of resources during listening” might be somewhat difficult. According to Kahneman and Frederick (2002), when faced with answering a difficult question (e.g., effort judgement), people answer an easier, substitute question, if a substitute attribute is highly accessible and reasonable. For effort judgements, some investigators have suggested participants use performance judgements as substitute attributes to make their ratings of effort (e.g., Moore and Picou, 2018), since judgements of word recognition performance are easy and accurate (Cox et al., 1991; Cienkowski and Speaks, 2000).

According to Kahneman and Frederick (2002), if the target attribute is accessible or if there is no reasonable alternative substitute, people would be less likely to use a heuristic. Thus, instead of using language that includes the words “effort” or “work,” it might be possible to use language that elicit judgements of “effort” that align with behavioral indices of listening effort. In adults, Picou et al. (2017b) and Picou and Ricketts (2017) identified that asking participants to judge the extent to which they wanted to control the listening situation (“want to do something to improve the situation, such as move to a quiet room or ask the talker to speak up”) elicited subjective ratings that were more highly correlated with responses times in a dual-task paradigm than did asking participants “how hard” they had to work or how “tired” they were. That is, the desire to control the situation was a target attribute that was easy to answer and yet was still associated with behavioral listening effort.

Reports of subjective ratings of effort from school-aged children are surprisingly scarce. The limited data available suggest that, as with adults, behavioral measures of listening effort and subjective ratings can be discrepant (Hicks and Tharpe, 2002; Gustafson et al., 2014). For example, Gustafson et al. (2014) reported that digital noise reduction in hearing aids improved ratings of clarity and reduced listening effort (measured behaviorally using verbal response times), though the two outcomes were not correlated. Based on the findings in adults, it might be possible to use language in the ratings task to elicit responses from school-aged children that align with behavioral indices of listening effort. However, the questions used by Picou et al. (2017b) are likely not appropriate for school-aged children.

For the current study, the questions established by Picou et al. (2017b) were modified for language and content. Specifically, to evaluate a target attribute of “control,” the question was reworded to have participants rate the degree to which they wanted to “turn up the lady’s voice” (the study stimuli were spoken by a female talker). This question was a simpler version of the question used previously.

In addition to modifying the control question, a new question was developed to assess children’s perception of the passage of time (i.e., “how long did that feel”). The sense of time passing is complex and multidimensional, but in some circumstances can be affected by cognitive load (Khan et al., 2006; Block et al., 2010). For example, in adults, simple laboratory tasks are perceived as taking longer than tasks that require deeper processing (Sucala et al., 2011). If someone is investing more resources during a listening task, fewer resources would be available for time awareness. Thus, if a task felt fast, it would indicate a participant was more cognitively engaged (exerting more listening effort) than if a condition felt slow. In total, the current study employed four subjective rating questions, two that are relatively straightforward, querying perceived performance and ease of listening, and two questions with the potential to associate with behavioral listening effort by probing related constructs, control and time.

A concept closely related to listening effort is mental fatigue. Fatigue is a multi-dimensional phenomenon that may be observed as a decrement in performance over time, or subjectively as a mood state, associated with feelings of tiredness, a lack of energy or motivation to continue on a task (Pichora-Fuller et al., 2016). Listening-related fatigue is thought to result, in part, from the application of sustained effort (Hornsby, 2013; Hornsby et al., 2016). However, evidence of fatigue as a result of sustained listening has not been empirically demonstrated in school-aged children. Two studies have evaluated potential fatigue in this population, both using a scale described by Bess and Hornsby (2014). The scale, referred to here as the “Right Now Fatigue Scale” is administered at various times throughout a test session and asks a participant to rate how they feel “right now” on five questions. The questions probe the degree to which a participant feels tired, that the task is easy, they are able to focus, they have trouble thinking, or their head hurts.

McGarrigle et al. (2017) used the survey, in addition to response-time and pupillometry indices, to evaluate listening-related fatigue and effort in two environments. The environments reflected a “typical” classroom with a poor SNR and an “ideal” classroom with a more favorable SNR. Outcomes were the same on all tasks after listening in both rooms. In addition, ratings of fatigue were generally low, suggesting participants did not experience listening-related fatigue. However, participants completed the Right Now Fatigue Scale only at the end of testing in each environment. It is possible listening-related fatigue would have been evident as a change in fatigue ratings relative to a pre-test score. In addition, the authors only analyzed a total fatigue score, calculated as the mean response to all five questions. It is not clear if all five questions are equally sensitive to listening-related fatigue.

Another study using the Right Now Fatigue Scale provides indirect evidence of listening-related fatigue. Bess and Hornsby (2014) reported descriptive changes in self-reported fatigue using mean scores from all five questions obtained at several time points throughout the course of a research visit lasting 2.5 to 3 h. Although fatigue scores were generally low, the authors described increased fatigue over the duration of the research visit, which included both active and passive listening tasks. However, the changes in fatigue were small and not analyzed statistically. Thus, it remains unclear if sustained, active listening affects fatigue in school-aged children. Furthermore, like McGarrigle et al. (2017), Bess and Hornsby only reported mean responses to all five questions on the scale. It is possible changes in fatigue would be larger with some questions (e.g., related to tiredness or task ease) than other questions (e.g., related to trouble thinking or head hurting). As noted above, the relative sensitivity of the five Right Now Fatigue Scale questions to listening-related fatigue have not been previously evaluated.

The purpose of this study was three-fold. The primary purpose was to evaluate the effects of noise and moderate reverberation on listening effort in school-aged children with normal hearing. Based on FUEL, it was hypothesized that noise and moderate reverberation would increase listening effort as evidenced by slower response times during a dual-task paradigm and by subjective ratings. It was also expected that the effects of noise would be larger when the reverberation time was longer. A second purpose was to evaluate the relationship between subjective and behavioral measures of listening effort, with specific interest in questions that reconcile the noted discrepancy between behavioral and subjective indices. It was hypothesized that questions related to time and a desire to control the situation would be related to behavioral listening effort and questions related to performance and listening ease would be related to speech recognition scores. A third purpose was to evaluate the effect of reverberation on self-reported fatigue, taking into consideration the limitations of previous studies, notably the inclusion of a pre-test rating, evaluating fatigue after sustained, active listening, and analyzing responses to self-report questions separately. It was expected that the change in fatigue would be higher after sustained listening in moderate, compared to low, reverberation.

## Materials and Methods

### Participants

Twenty school-aged children (five males) participated in the study (aged 10 to 17 years, *M* = 13.25, *SD* = 2.34). Participants were recruited via word of mouth and via e-mail solicitation to people who have opted in to receive e-mail notifications regarding research participation opportunities. All participants had normal hearing bilaterally, as evidenced by pure-tone, air conduction thresholds of 20 dB HL or better. In addition, all participants exhibited normal middle ear function on the day of testing, as indicated by normal middle ear pressure and compliance measured with 226 Hz tympanometry. Based on participant and parent/guardian self-report, all participants were typically developing with no known neurological, cognitive, vision, or developmental disorders.

All participants underwent speech in noise testing using the Bamford-Kowal-Bench, Speech in Noise test (BKB-SIN; Etymotic Research, 2005). The purpose of this test was to evaluate a participant’s speech understanding in noise ability in order to establish the SNR to be used for the listening effort and fatigue procedures. For the listening effort and fatigue procedures, it was desirable to target specific performance levels (described below). The use of the BKB-SIN procedures allowed for setting of individualized SNRs without using the same stimuli that would be used later for experimental testing. Pilot testing was used to establish the relationship between BKB-SIN scores and SNRs necessary to approximate 84%- (easy) and 77%- (moderately difficult) word recognition performance with the experimental stimuli. Participants for pilot testing included adults and children (10–17 years old) with normal hearing bilaterally; these participants were not otherwise involved with the study.

Testing with BKB-SIN was accomplished bilaterally through supra-aural headphones (TDH-50) using standard test instructions in an audiometric sound booth. One passage pair was used for each participant. A passage pair consists of 10 sentences spoken by a male talker presented in a four-talker babble background noise. The SNR is progressively decreased in increments of 3 dB after each sentence. The starting SNR is +21 dB and is progressively decreased in 3 dB steps to -6 dB. Specifically, the background noise level increases in 3 dB steps until the 8th sentence (0 dB SNR), for the remaining two sentences the level of the speech is decreased in 3 dB increments and the level of the noise is held constant. Consistent with test instructions, the level of the speech was set initially to be 70 dB HL (83 dB SPL). All stimuli during the BKBSIN test were routed from a compact disc player to an audiometer (Grason Stadler 61) and then to the headphones. After each sentence, the experimenter scored the number of keywords a participant correctly repeated back. Also based on test instructions, the SNR where participants were expected to understand 50% of speech (SNR-50) was calculated. SNR-50s recorded from study participants ranged from -2 to +4 dB (*M* = 0.5, *SD* = 1.54).

Procedures were approved by the Behavioral Sciences Committee at Vanderbilt University Medical Center’s Institutional Review Board (IRB # 180919). All participants gave written informed assent and parents/guardians provided written informed consent. Participants were paid an hourly rate; most testing was accomplished in a single test visit lasting approximately 2 h. This project was pre-registered with the Center for Open Science (osf.io/9dj2q).

### Behavioral Listening Effort

Behavioral listening effort was evaluated using a dual-task paradigm. The paradigm, described in detail by Picou et al. (2017a), included a primary task (monosyllable word recognition) and a secondary task (physical response to a visual probe). The monosyllable words, spoken by a female talker with an American English accent, were all nouns. The words were arranged into 8, 25-word lists based on pilot testing (completed with naïve adults with normal hearing). During presentation of the words, colored shapes (blue circle, blue triangle, yellow circle, or yellow triangle) were occasionally presented (18 out of 25 words). Participants’ secondary task was to respond as quickly as possible by pressing a touchscreen monitor when the correct color/shape combination was displayed (blue circle and yellow triangle) and to not touch the screen when the incorrect shapes were presented (blue triangle and yellow circle). They were instructed to repeat every word, regardless of the visual probe. Half of the shapes were probes (blue circle and yellow triangle) and half were foils or non-probes (blue triangle and yellow circle). The order of probe and non-probe trials was randomized across word lists. During the trials where no visual shape was displayed (7 out of 25 trials), a small white fixation cross (1 cm × 1 cm) was presented on a black screen. Colored shapes were approximately 6.5 by 6.5 cm and were also presented on a black screen.

### Subjective “Listening Effort”

Questions to elicit subjective ratings were developed for this study, each with a visual analog scale with verbal anchors at the end points. The questions were:

(1) How many words did you get right? (none of them – all of them);
(2) How easy was that? (not at all easy – very easy);
(3) How much did you want to turn up the lady’s voice? (not at all – a lot);
(4) How long did that feel? (it felt fast – it took forever).

An on-line survey was created with the four questions and four visual analog scales to facilitate data collection. The survey was presented to a participant after each condition using an internet-enabled tablet (Nexxus 7) with the survey visible. Participants responded to the questions in the same order using a response slider, which had 100 increments between the anchors. The response numbers were not visible to participants. Higher scores indicated participants rated their performance as higher, the task easier, had a stronger desire to turn up the talker’s voice, and had a longer perception of test time.

### Self-Reported Fatigue

All five questions from the Right Now Fatigue Scale were used to evaluate self-reported fatigue. The questions were described by Bess and Hornsby (2014) and were later used experimentally by McGarrigle et al. (2017). The questions are thought to relate to the constructs underlying fatigue. When answering the questions, participants were instructed to consider how they feel “right now.” Response options for all questions were “not at all (0),” “a little (1),” “some (2),” “quite a bit (3),” “a lot (4).” The questions were:

1- I feel tired;
2- It is easy for me to do these things;
3- My head hurts;
4- It’s hard for me to pay attention;
5- I have trouble thinking.

The anchor response options included schematic drawings of children experiencing the question response (e.g., a head down on the desk for “a lot” on the tired question). The complete survey is displayed in Appendix A of McGarrigle et al. (2017). For this study, the questionnaire was converted to an on-line survey, separate from the subjective rating survey. The response options were radio buttons. Surveys were presented to participants twice in a given test room (i.e., the low or moderately reverberant room). The first survey was given just prior to dual-task testing in a given room (“pre-test”) and again immediately following completion of all testing in the same room (“post-test”).

### Conditions

Participants completed dual-task testing and provided subjective ratings in six conditions, which varied by degree of reverberation (low and moderate) and background noise (quiet, easy, and moderately difficult). Testing in the low reverberation condition was completed in an audiometric test booth (T30 < 100 ms); testing in the moderate reverberation condition was completed in a moderately reverberant room (T30 = 834 ms). The T30 value is approximately equivalent to the RT60 measure; it is expressed as double the time it takes for energy to decay from 5 to 35 dB below the initial level (ISO 3382-1, 2009).

The background noise, when present, was a four-talker babble, as described in Picou et al. (2017a). Briefly, four female talkers simultaneously read sentences from the Connected Speech Test (Cox et al., 1987). Each talker’s voice originated from a single loudspeaker. The loudspeaker location of the talker changed after each sentence. The same sentence was never read by two talkers at the same time.

The background noise conditions were achieved by varying the level of the noise. In quiet, no background noise was present. In the other conditions, the level of background noise was chosen relative to a participant’s BKB-SIN SNR-50 score to create an “easy” and a “moderately difficult” test condition. The use of individualized SNRs based on a participant’s speech understanding in noise abilities ensured participants were listening in a performance range where the listening effort task would be sensitive to changes in SNR. Previous work demonstrates that response times during listening effort tasks exhibit an inverse U-shaped function (Wu et al., 2016), where response times progressively increase until a point of cognitive overload where participants exert less effort because cognitive demands exceed cognitive resources (e.g., Granholm et al., 1996; Zekveld et al., 2014). According to Wu et al. (2016), in adults, response times peak around 30–50% correct performance levels. It was desirable in this study to keep performance in a range where changes in SNR would not result in response times in the cognitive overload section of the performance-intensity function. Thus, word recognition performance levels were targeted to be 84 and 77% correct. Based on the aforementioned pilot testing, the “easy” condition was a SNR set to be 5 dB less favorable than the participant’s BKB-SIN score. The mean noise level in the “easy” condition, hereafter referred to as the SNR84 condition, was 69.5 dB. The “moderately difficult” condition was a SNR set to be 9 dB less favorable than the participant’s BKB-SIN score. The average background noise level in this condition, hereafter referred to as SNR77, was 73.5 dB SPL. The speech was always 65 dB SPL, resulting in mean SNRs of -4.5 and -8.5 dB for the SNR84 and SNR77 conditions, respectively.

### Test Environment

In a sound booth (4 m × 4.3 m × 2.7 m), participants provided assent, a parent/guardian provided informed consent, and a researcher completed tympanometry, hearing testing, and BKB-SIN testing. In addition, dual-task testing and subjective ratings comprising the low reverberation conditions (T30 < 100 ms) were completed. Speech signals were presented via custom programming of experimental software (Presentation v 14, Neurobehavioral Systems), routed through an audiometer (Madsen Orbiter 922 v2), to a loudspeaker (Bowers and Wilkins 685 S2) 1.25 m in front of a listener (0°). The four background noise channels were presented via sound editing software (Adobe Audition CSS5) and a multichannel sound card (Layla Echo), to an amplifier (Russound DPA-6.12), and finally to loudspeakers (Bowers and Wilkins 685 S2). The loudspeakers were 1.25 m from the participant and were placed at 45, 135, 225, and 315°.

Dual-task testing was also completed in a moderately reverberant room (5.5 m × 6.5 m × 2.25 m), which has solid, random-incidences, walls and ceilings, and a concrete floor. Unoccupied and untreated, the T30 of this test space is approximately 2100 ms. Floor carpet and four ceiling acoustic blankets (Sound Spotter 124, 4 × 4) were used to limit reverberation to the desired level (T30 = 834 ms). During testing, the speech was presented from a separate control room via custom programming of experimental software (Presentation v 12.0, Neurobehavioral Systems) and was routed to a self-powered loudspeaker (Tannoy 600A) 1.25 m in front of a participant (0°). The noise was routed from sound editing software (Adobe Audition v1.5) and a multichannel sound card (Layal Echo) through an amplifier (Crown) and to the four noise loudspeakers (Tannoy System 600). The loudspeakers were located 3.5 m from the participant at 45, 135, 225, and 315°. In both rooms, visual probes were displayed on a touchscreen monitor (Dell S2240T) placed directly in front of a participant. The monitor accepted touch responses via USB cable connected to the experimental control computer.

### Procedures

Table 1 indicates the procedural order and approximate test time for study tasks. After informed consent and assent procedures, a participant underwent hearing and immittance testing using standard clinical procedures. Then, they completed dual-task testing in one of the two rooms. In a given room, participants first completed three practice conditions: (1) secondary-task only in quiet, (2) primary and secondary tasks combined in quiet, (3) primary and secondary tasks combined in background noise with a favorable SNR (1 dB less favorable than a participant’s SNR-50 with expected word recognition performance of 98%, hereafter labeled SNR98). Immediately following these three practice conditions, participants performed the secondary task only in quiet again. This served as their room-specific baseline. Following these four conditions, participants completed the self-report fatigue questionnaire (pre-test fatigue). Then, each participant completed dual-task procedures in a given SNR. Following each 25-word list of dual-task testing in a given condition, the participant answered the four subjective ratings questions, answering the questions about their experience during the dual-task testing. Condition order (quiet, SNR84, and SNR77) within a room was randomized across participants. Each condition was tested twice; the second round of condition testing was initiated immediately after the first round was completed. After testing was completed in one room, participants answered again the five fatigue questions (post-test fatigue). Testing in a given room lasted approximately 25 min and breaks were discouraged during testing. After testing was fully completed in a room, participants took a 15-min break and switched rooms. Test order of rooms was counterbalanced across participants; half were tested in the low reverberant room first.

**Table 1:** Order of study procedures.

				Number of words or	Approximate
	Procedure	Task	SNR	questions	time (min)
1	Informed consent and assent				10
2	Audiometric evaluation and BKBSIN testing				10
3	Practice 1	Secondary task only	Quiet	25	3
4	Practice 2	Dual tasks	Quiet	25	3
5	Practice 3	Dual tasks	SNR98	25	3
6	Baseline	Secondary task only	Quiet	25	3
7	Fatigue survey pre-test	Questionnaire		5	2
8	Condition 1a	Dual tasks	Quiet, SNR84, or SNR77	25	3
9	“Listening effort” survey 1a	Questionnaire		4	1
10	Condition 2a	Dual tasks	Quiet, SNR84, or SNR77	25	3
11	“Listening effort” survey 2a	Questionnaire		4	1
12	Condition 3a	Dual tasks	Quiet, SNR84, or SNR77	25	3
13	“Listening Effort” Survey 3a	Questionnaire		4	1
14	Condition 1b	Dual tasks	Quiet, SNR84, or SNR77	25	3
15	“Listening effort” survey 1b	Questionnaire		4	1
16	Condition 2b	Dual tasks	Quiet, SNR84, or SNR77	25	3
17	“Listening effort” survey 2b	Questionnaire		4	1
18	Condition 3b	Dual tasks	Quiet, SNR84, or SNR77	25	3
19	“Listening effort” survey 3b	Questionnaire		4	1
20	Fatigue survey post-test	Questionnaire		5	1
21	Break				15
22	Repeat procedures 3–20 in the second room				40

*Conditions were repeated twice within each level of reverberation (indicated by “a” and “b” below). Detailed procedures reflect testing in the first level of reverberation, which were then repeated in the second level of reverberation following the break.*

### Data Analysis

Prior to analysis, word recognition scores were converted to rationalized arcsine units (RAU) according to the equations in Studebaker (1985). Word recognition scores, response times, and subjective ratings were evaluated separately using generalized linear models with two factors of interest: SNR (quiet, SNR84, and SNR77) and reverberation (low and moderate) and participant as a random factor. The relationship between response times and subjective ratings was explored using partial correlation analyses, statistically controlling for SNR (quiet, SNR84, or SNR77). In the correlation analyses, data were pooled across conditions; no correction was made to account for multiple data points from the same participant. Responses to the five self-reported fatigue questions were analyzed as a single score based on a participant’s mean response to all five questions (with responses to question two reversed). In addition, questions were analyzed separately because it was not clear which, if any, of the questions would be sensitive to fatigue. In all cases, responses were analyzed using a generalized linear model with two factors of interest (pre-test/post-test, low reverberation/moderate reverberation). All analyses were conducted with IBM SPSS Statistics 25.

## Results

### Word Recognition Performance

**FIGURE 1:**
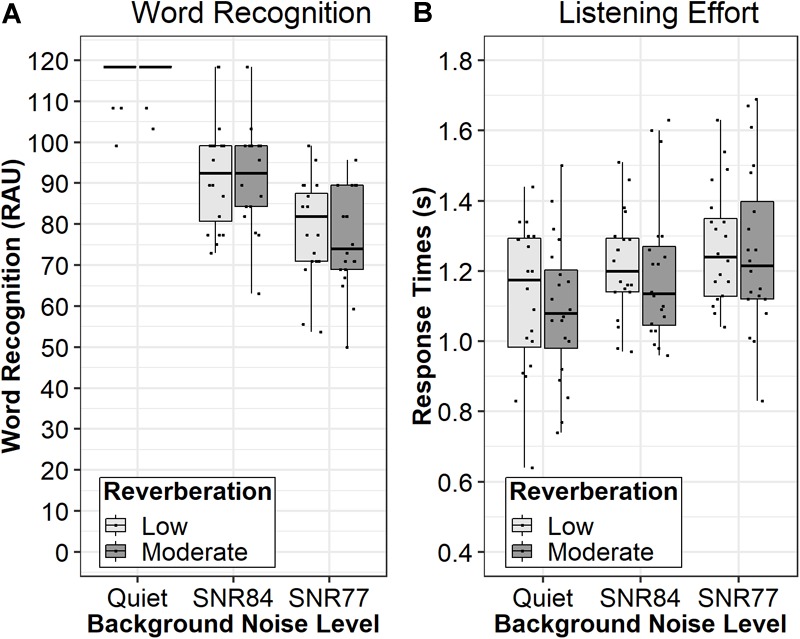
Median word recognition performance (RAU; **A**) and behavioral listening effort (sec; **B**) for each background noise condition. Boxes represent the 1st through 3rd quartile. Light gray boxes reflect scores in low reverberation (T30 < 100 ms) and dark gray boxes reflect scores in moderate reverberation (T30 = 834 ms).

Analysis of the transformed word recognition scores collected during the dual-task paradigm, displayed in Figure 1A (left panel), revealed a significant main effect of SNR [*F*(2,73.37) = 231.70, *p* < 0.0001, $\eta_{p}^{2}$ = 0.86]. The main effect of Reverberation (*p* = 0.62, $\eta_{p}^{2}$ = 0.002) and the Reverberation × SNR interaction (*p* = 0.68, $\eta_{p}^{2}$ = 0.01) were non-significant. The mean difference in performance between the low and moderate reverberation conditions was 0.94 RAU (95% CI: -2.79 to 4.67). Results of follow-up pairwise comparison testing, displayed in Table 2, revealed word recognition performance was significantly different in all SNRs (*p* < 0.001). These data demonstrate adding background noise and increasing the background noise both significantly reduced word recognition performance, but increasing reverberation did not affect performance.

**Table 2:** Mean differences between background noise conditions (quiet, SNR84, and SNR77) with each of the outcomes (word recognition, response times, and four subjective ratings).

	Word recognition	
	scores (RAU)			Response times (ms)	
	SNR84	SNR77		SNR84	SNR77
**Quiet**	-24.94	-38.94	**Quiet**	98.10	155.61
*p*-Value	<*0.001*	*<0.001*	*p*-Value	*0.025*	*.001*
95% CI	-20.75 to 29.13	-34.87 to -43.01	95% CI	12.40 to 183.80	64.54 to 246.69
**SNR84**		-14.00	**SNR84**		57.51
*p*-Value		<*0.0001*	*p*-Value		*0.183*
95% CI		-8.58 to -19.43	95% CI		027.71 to 142.74

	**Ratings of performance**			**Ratings of control**	
	**SNR84**	**SNR77**		**SNR84**	**SNR77**

**Quiet**	-15.34	-25.86	**Quiet**	37.40	49.48
*p*-Value	<*0.0001*	*<0.0001*	*p*-Value	<*0.0001*	*<0.0001*
95% CI	-8.98 to -21.70	-18.78 to -32.95	95% CI	27.71 to 47.09	39.86 to 59.09
**SNR84**		-10.53	**SNR84**		12.08
*p*-Value		*0.013*	*p*-Value		*0.012*
95% CI		-2.27 to -18.78	95% CI		2.77 to 21.38

	**Ratings of ease**			**Ratings of time**	
	**SNR84**	**SNR77**		**SNR84**	**SNR77**

**Quiet**	-25.94	-39.60	**Quiet**	14.80	21.06
*p*-Value	<*0.0001*	*<0.0001*	*p*-Value	<*0.0001*	*<0.0001*
95% CI	-16.72 to -35.16	-30.16 to -49.04	95% CI	5.16 to 24.44	12.56 to 29.56
**SNR84**		-13.66	**SNR84**		6.26
*p*-Value		*0.013*	*p*-Value		*0.236*
95% CI		-2.98 to -24.34	95% CI		-4.20 to 16.72

*Negative values indicate scores in quiet were higher than scores in noise or that scores in SNR84 were higher than scores in the SNR77 condition. Actual *p*-Values and 95% CI of the difference are also provided.*

### Behavioral Listening Effort

Mean baseline response times were 1036.8 ms (std. error = 47.46) and 1099.3 ms (std. error = 53.9) in the moderate and low reverberant conditions, respectively. They were not significantly different from each other [*F*(1,37.41) = 0.76, *p* = 0.39]. Analysis of the response times during the dual-task paradigm, displayed in Figure 1B (right panel), revealed a significant main effect of SNR [*F*(2,69.88) = 5.94, *p* < 0.005, $\eta_{p}^{2}$ = 0.15]. The main effect of Reverberation (*p* = 0.57, $\eta_{p}^{2}$ = 0.003) and the Reverberation × SNR interaction (*p* = 0.97, $\eta_{p}^{2}$ < 0.001) were non-significant. The mean difference in performance between the low and moderate reverberation conditions was 20.5 ms (*p* = 0.57, 95% CI: -50.4 to 91.4). Results of follow-up pairwise comparison testing, displayed in Table 2, revealed significant response time differences only between quiet and noise conditions (*p* < 0.05). Taken together, these data demonstrate the addition of background noise increased behavioral listening effort, but further increases in background noise level or increased reverberation did not increase behavioral listening effort.

### Subjective “Listening Effort”

#### Performance

**FIGURE 2:**
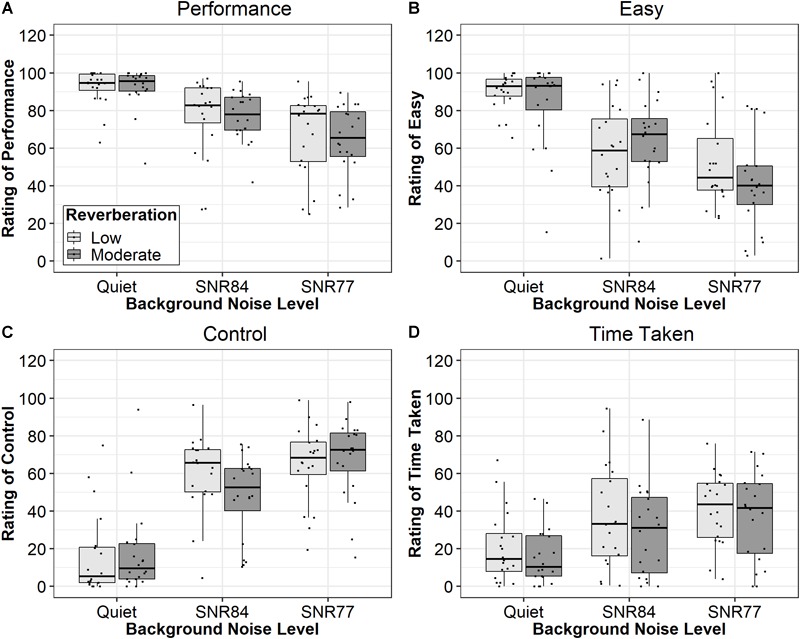
Median subjective ratings of performance (top left panel **A**), ease of listening (bottom left panel **B**), control (top right panel **C**), and time (bottom right panel **D**) for each SNR. Boxes represent the 1st through 3rd quartiles. Light gray boxes reflect scores in low reverberation (T30 < 100 ms) and dark gray bars reflect scores in moderate reverberation (T30 = 834 ms).

Ratings of performance (how many words did you get right?) are displayed in Figure 2A (top left panel). Analysis revealed a significant main effect of SNR [*F*(2,62.99) = 31.69, *p* < 0.0001, $\eta_{p}^{2}$ = 0.50]. The main effect of Reverberation (*p* = 0.71, $\eta_{p}^{2}$ < 0.01) and the Reverberation × SNR interaction (*p* = 0.13, $\eta_{p}^{2}$ < 0.01) were not significant. The mean difference in ratings between the low and moderate reverberation conditions was 1.09 (95% CI: -4.81 to 6.99). Follow-up pairwise comparison testing results, displayed in Table 2, revealed ratings of performance were significantly different in all SNRs (*p* < 0.001). This pattern of results is the same as the pattern of results for word recognition performance.

#### Ease of Listening

Ratings of ease of listening (how easy was that?) are displayed in Figure 2B (bottom left panel). Analysis results revealed a significant main effect of SNR [*F*(2,66.94) = 39.45, *p* < 0.0001, $\eta_{p}^{2}$ = 0.54]. The main effect of Reverberation (*p* = 0.38, $\eta_{p}^{2}$ < 0.01) and the Reverberation × SNR interaction (*p* = 0.23, $\eta_{p}^{2}$ = 0.04) were non-significant. The mean difference in ratings between the low and moderate reverberation conditions was 3.52 (95% CI: -0.41 to 11.50). Follow-up pairwise comparison testing results, displayed in Table 2, revealed ratings of ease were significantly different in all SNRs (*p* < 0.05). This pattern of results is the same as the pattern of results for word recognition performance and perceived performance.

#### Control

Ratings of control (how much did you want to turn up the lady’s voice?) are displayed in Figure 2C (top right panel). Analysis results revealed a significant main effect of SNR [*F*(2,77.61) = 55.87, *p* < 0.0001, $\eta_{p}^{2}$ = 0.50]. The main effect of Reverberation (*p* = 0.52, $\eta_{p}^{2}$ < 0.01) and the Reverberation × SNR interaction (*p* = 0.20, $\eta_{p}^{2}$ = 0.04) were non-significant. The mean difference in ratings between the low and moderate reverberation conditions was 2.50 (95% CI: -5.25 to 10.25). Follow-up pairwise comparison results, displayed in Table 2, revealed ratings of control were significantly different in all SNRs (*p* < 0.05). This pattern of results is the same as the pattern of results for word recognition performance, perceived performance, and ease of listening ratings.

#### Time

Ratings of a listener’s sense of time (how long did that feel?) are displayed in Figure 2D (bottom right panel). Analysis results revealed a significant main effect of SNR [*F*(2,79.71) = 13.31, *p* < 0.001, $\eta_{p}^{2}$ = 0.25]. The main effect of Reverberation (*p* = 0.15, $\eta_{p}^{2}$ = 0.02) and the Reverberation × SNR interaction (*p* = 0.94, $\eta_{p}^{2}$ < 0.01) were not significant. The mean difference in ratings between the low and moderate reverberation conditions was 5.65 (95% CI: -2.12 to 13.42). Follow-up pairwise comparison results, displayed in Table 2, revealed ratings of giving up were significantly different in the noise conditions compared to quiet condition (*p* < 0.01). This pattern of results is the same as the pattern of results for response times during the secondary task.

### Relationship Between Variables

Partial correlations were conducted between word recognition scores (RAU), response times (ms), and responses to each of the four questions while controlling for test SNR. Results, displayed in Table 3, reveal that the word recognition performance was significantly correlated with ratings of performance, ease of listening, and control [*r*(157) = 0.18 to 0.26], in addition to response times [*r*(157) = 0.44]. Word recognition performance was not correlated with ratings of time. Response times were correlated only with word recognition performance and with ratings of time [*r*(157) = 0.17]. These data demonstrate ratings of time are related to response times, whereas ratings of control, ease of listening and perceived performance are related to word recognition performance.

**Table 3:** Partial correlation coefficients (and *p*-Values in parentheses) examining the relationships between word recognition performance (RAU), response times during the secondary task (ms), and ratings of ease of listening, control, and time, while controlling for condition (quiet, SNR84, and SNR77).

	Word recognition	Performance	Ease	Control	Time
Response times	-0.44 (<*0.001*)	-0.15 (*0.06*)	-0.09 (*0.29*)	0.001 (*0.99*)	0.17 (*0.03*)
Word recognition		0.26 (<*0.01*)	0.18 (*0.02*)	-0.22 (<*0.01*)	-0.14 (*0.08*)
Performance			0.48 (<*0.001*)	-0.44 (<*0.001*)	-0.11 (*0.19*)
Ease				-0.61 (<*0.001*)	-0.34 (<*0.001*)
Control					0.25 (<*0.01*)

*Ratings were on a 100-point scale. For all correlations, *n* = 160 and *df* = 157.*

To evaluate the accuracy of subjective ratings of performance, a repeated measures analysis of variance was conducted with three within-participant factors: outcome variable (word recognition performance in percent correct, rating of perceived accuracy), reverberation (low and moderate), and SNR (quiet, SNR84, and SNR77). This analysis was not planned *a priori* and thus not included in the pre-registration. Results indicated a significant main effect of outcome [*F*(1,19) = 15.20, *p* < 0.01, $\eta_{p}^{2}$ = 0.44] and a main effect of SNR [*F*(2,18) = 34.48, *p* < 0.001, $\eta_{p}^{2}$ = 0.79]. The main effect of Reverberation and all the interactions were non-significant (*p* > 0.50). These results demonstrate participants underestimated their word recognition performance (*M* = 9.69, 95% CI: 4.49 to 14.89), but the magnitude of the underestimation was consistent across conditions. That is, across conditions, participants rated their performance as 9.69 percentage points lower than their actual word recognition performance.

### Self-Reported Fatigue

**FIGURE 3:**
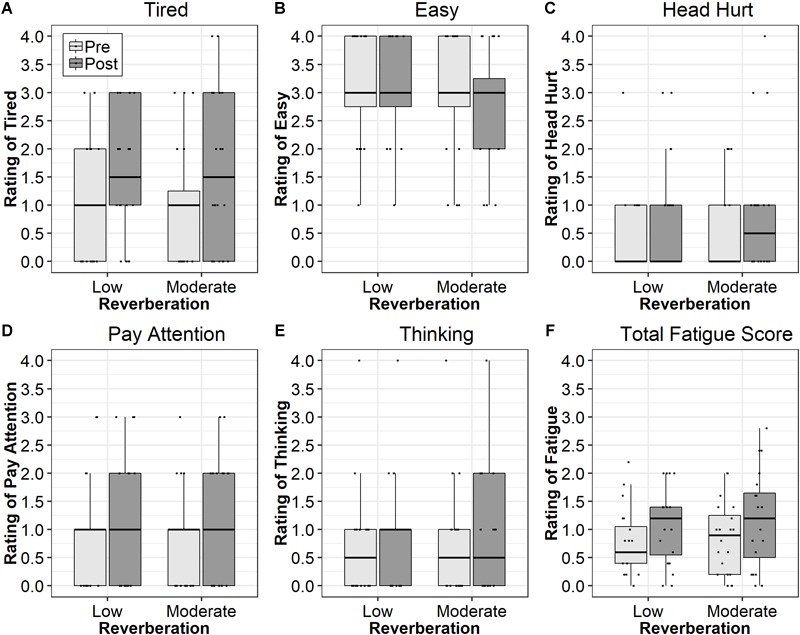
Median responses to the self-reported fatigue questions related to feeling tired **(A)**, task ease **(B)**, head hurting **(C)**, paying attention **(D)**, difficulty thinking **(E)**, and total fatigue score **(F)**. Boxes represent the 1st through 3rd quartiles. Light gray boxes reflect scores in low reverberation (T30 <100 ms) and dark gray boxes reflect scores in moderate reverberation (T30 = 834 ms).

Mean responses to all five self-reported fatigue questions, in addition to the mean fatigue score (with question two reversed) are displayed in Figure 3. When the mean of all five responses was used to indicate self-reported fatigue, analyses revealed a significant main effect of Time [*F*(1,85.73) = 4.86, *p* < 0.05, $\eta_{p}^{2}$ = 0.05] and no significant effect of Reverberation (*p* = 0.86, $\eta_{p}^{2}$ < 0.001) or Reverberation × Time interaction (*p* = 0.90, $\eta_{p}^{2}$ < 0.001). The mean difference between pre- and post-test was 0.3 points (95% CI: 0.03 to 0.57), a 35.8% increase relative to pre-test ratings. Analysis of the question about tiredness revealed a significant main effect of Time [*F*(1,71.05) = 4.90, *p* < 0.05, $\eta_{p}^{2}$ = 0.06], a 56.1% increase in reported tiredness relative to pre-test ratings. The main effect of Reverberation (*p* = 0.92, $\eta_{p}^{2}$ < 0.01) and the Reverberation × Time interaction (*p* = 0.92, $\eta_{p}^{2}$ < 0.01) were not significant. These results demonstrate ratings of tiredness were significantly higher after sustained listening (*M* difference = 0.58). None of the other questions resulted in ratings that were significantly different in the post-test compared to the pre-test (*p* > 0.10, $\eta_{p}^{2}$ < 0.03). The mean differences between the pre- and post-tests ranged from 0.18 to 0.35 points. These data indicate increases in self-reported fatigue resulting from a sustained listening task, as measured by the overall score and by rating of tiredness, was independent of level of reverberation. Exploratory analysis with an additional variable, test order (first room versus second room), revealed an identical pattern of results, suggesting test order did not affect ratings of fatigue.

## Discussion

The purpose of this project was three-fold: (1) to evaluate the effects of noise and reverberation on behavioral listening effort and subjective ratings of performance, ease of listening, desire to control, and perception of time, (2) to evaluate the relationship between behavioral and subjective indices of listening effort and (3) to evaluate the effects of reverberation on self-reported listening-related fatigue. Each purpose will be considered in turn.

### Effects of Noise and Reverberation on Listening Effort

Based on FUEL and ELU, it was expected that background noise and reverberation would both increase listening effort. However, the current results do not fully confirm this hypothesis. Although the addition of background noise increased listening effort, behavioral listening effort was the same in the low (T30 < 100 ms) and moderate reverberation conditions (T30 = 834 ms). There are a number of possible explanations that could account for the non-significant findings.

**FIGURE 4:**
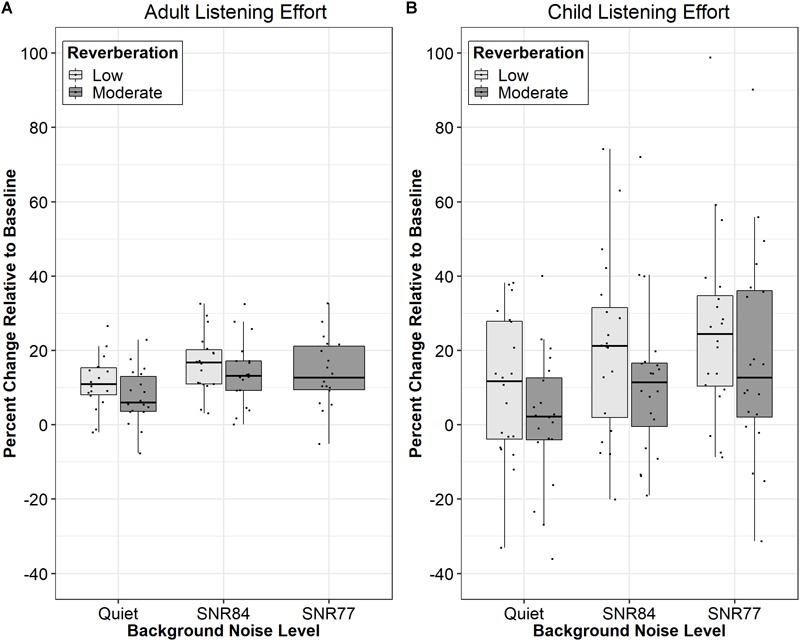
Comparison of data from young adults (replotted data from Picou et al., 2016; **A**) and school-aged children **(B)**. Median percent listening effort relative to baseline is displayed where the response time during dual-task testing (RT_Dual_Task) is reflected as the percent increase relative to baseline testing (RT_Baseline). Specifically, percent listening effort is calculated as 100^∗^(RT_Dual_Task-RT_Baseline)/RT_Baseline. Boxes represent the 1st through 3rd quartiles.

First, it is possible the dual-task was not sensitive to changes in reverberation, as dual-task results in children might be less valid compared to other methodologies (Choi et al., 2008; McGarrigle et al., 2019), unlike in adults where dual-task paradigms are accepted measures of behavioral effort (e.g., Gagne et al., 2017). To compare the results of this study with the results of an earlier study with young adults (Picou et al., 2016), percent change in listening effort was calculated using the following formula:

(1) $$Percent\;Listening\;Effort=\;\frac{100*(RT_{\text{Dual}\_\text{task}}-RT_{\text{Baseline}})}{RT_{\text{Baseline}}}$$

where RT_Dual_Task_ is the secondary task response time in a given condition and RT_Baseline_ is the secondary task response time without the primary task. Figure 4 displays percent listening effort for adults (Picou et al., 2016) and school-aged children (current study). For both groups, introducing background noise increased listening effort, whereas increasing the background noise and increasing reverberation time did not increase listening effort. Thus, the pattern of results with the school-aged children, although more variable, was similar to the findings in adults.

Second, the results of the study are limited to the specific acoustic conditions evaluated, which include relatively short stimuli (words rather than sentences or passages), SNRs that resulted in good word recognition performance (mean lowest performance 77%), a relatively small test room (moderate reverberation room was approximately 80 m^3^), and a speaker and listener inside the critical distance. Larger rooms are more likely to have longer reverberation times (Knecht et al., 2002) and potentially more detrimental reverberation effects because late reflections comprise a greater portion of the total reverberant energy. Furthermore, if a listener is outside the critical distance from a loudspeaker, or the distance at which reverberant and direct energy are equivalent (Peutz, 1971; Egan, 1988), reverberant energy will dominate the signal, potentially increasing the effects of reverberation on listening effort. These acoustic factors warrant consideration in future work.

Third, and related, the moderate reverberation time was only 834 ms. For the school-aged children in this study, this level of reverberation was insufficient to affect word recognition performance. Thus, it might not be expected to affect listening effort either. Interestingly, Picou et al. (2016) used the same reverberation time with the young adults whose data are presented in Figure 4. In the earlier study, moderate reverberation did reduce word recognition performance, whereas it did not for the children in the current study. The reason for the discrepancy is not clear and might be related to the increased variability in the school-aged children or to the typical experiences of children, who routinely listen in reverberant classroom environments. Regardless of the explanation, it seems clear that future work is necessary to evaluate the limits of the non-significant reverberation effects on listening effort.

Fourth, participants were tested in reverberant rooms and were permitted to move their heads during testing. Conversely, investigators who previously demonstrated increases in listening effort with increased reverberation used recorded signals convolved with impulse room responses (Sato et al., 2008, 2012; Rennies et al., 2014). This methodology allows for testing across a wide range of reverberation times in a controlled manner, but unnaturally eliminates head movements. Head movements can help listeners resolve ambiguous cues (Wallach, 1939, 1940) and improve their SNR (Grange and Culling, 2016). Thus, it is possible that in real rooms, the negative consequences of reverberation on listening could also be alleviated with head movements.

Fifth, the participant age range was large (10–17 years). It is possible the effects of reverberation on listening effort are more likely to be evident in one group of listeners than another, although it is not clear which group of listeners might be more likely to demonstrate changes in effort with increased reverberation. Relative to older children, younger children are more likely to demonstrate worse speech recognition performance in noise (Klatte et al., 2010b; Neuman et al., 2010) and in reverberation (Neuman and Hochberg, 1983), so they might also be more vulnerable to the effects of noise and reverberation on listening effort. Conversely, the younger children tend to be more variable on some measures of listening effort (Picou et al., 2017a) and the additional variability might limit the possibility of demonstrating significant effects of reverberation on listening effort. Exploratory analysis with the current data set revealed a similar pattern of results with children when divided into four age groups (10–11, 12–13, 14–15, and 16–17 years). However, the sample size in each age group precludes full investigation into the developmental effects of reverberation or SNR on listening effort, warranting future study.

Finally, it is possible that moderate reverberation does not increase behavioral listening effort, contrary to the expectations outlined in the existing frameworks. This final possibility is based on converging lines of emerging evidence, such as increased listening effort with the addition of acoustic paneling (Amlani and Russo, 2016), non-significant effects with behavioral paradigms (Picou et al., 2016; Peng and Wang, 2019), and differential physiological effects of noise and distortion (Francis et al., 2016). In some cases, the reverberation affected word recognition performance but did not have a comparable detrimental effect on listening effort (Peng and Wang, 2016, 2019; Picou et al., 2016). If future studies continue to demonstrate results contrary to framework predictions, it will be necessary to update the FUEL (Pichora-Fuller et al., 2016) and ELU framework (Rönnberg et al., 2013). Perhaps factors that affect signal transmission, such as noise and reverberation, should be considered separately, rather than assuming that all interferers with signal transmission increase listening effort.

Alternatively, the frameworks may need to be clarified to include the possibility that the long-term memory stores against which incoming speech signals are compared do not exclusively represent clean memory traces. Instead, it is possible that with experience (e.g., listening in classrooms), listeners can update or expand long-term memory representations to include distorted versions of speech. This possibility is consistent with an episodic theory of lexical access, which suggests perceptual details of speech (e.g., talker gender, speaking rate) are encoded in memory along with linguistic information (e.g., Goldinger, 1998; Grossberg, 2003) and with the observed effects of experience with reverberant stimuli (e.g., Zahorik and Brandewie, 2016). These hypotheses are speculation beyond the scope of this article, but they warrant further investigation.

### Subjective Ratings of “Listening Effort”

A second purpose of this study was to evaluate the relationship between subjective and behavioral indices of listening effort in school-aged children. The results of the current study demonstrate that children’s responses of perceived performance are significantly related to their ratings of actual performance. These data are consistent with findings in adult listeners, whose rated and actual performance are highly correlated (Cox et al., 1991; Cienkowski and Speaks, 2000; Saunders et al., 2004). Somewhat unlike adult listeners with normal hearing whose perceived and actual performances are nearly identical (e.g., Cox et al., 1991; Saunders et al., 2004), the school-aged children in this study consistently underestimated their performance by approximately 10 percentage points. This might reflect a lack of confidence in their understanding ability or the measurement methodology. The visual analog scale used for collecting subjective ratings included verbal anchors at the end points; no numbers were provided along the scale. Thus, participants were blinded to the score they were reporting.

The results of the current study also demonstrate that ratings of “ease of listening” are more closely related to actual and perceived performance than to the behavioral measure of listening effort. This finding is consistent with the adult literature dissociation between ratings of “ease” or “effort” and behaviorally measured listening effort (Feuerstein, 1992; Hicks and Tharpe, 2002; Lemke and Besser, 2016). As suggested by these authors, among others, the emerging pattern of results discourage investigators from using “ease of listening” as a proxy for behavioral listening effort.

Instead of ease of listening, it was expected that a question related to desire to control the situation (turn up the lady’s voice) would relate to behavioral listening effort, consistent with ratings of control in adults (Picou and Ricketts, 2017; Picou et al., 2017b). However, ratings of control revealed a pattern of results identical to those of word recognition performance, ratings of performance, and ratings of ease, suggesting participants were using their performance as a basis for rating their desire to control the listening situation. Because self-control has been related to willingness to accept background noise (Nichols and Gordon-Hickey, 2012), it is possible the difference between the results for children and adults is related to the development and understanding of self-control. It is also possible that ratings of control are affected differentially in quiet and in noise, where overall level of the speech might contribute to ratings in quiet but noise level dominates ratings in noise. Regardless of the explanation, it appears ratings of control were not an effective indirect, subjective measure of behavioral listening effort for children in this study.

Instead, subjective ratings of time perception were the only ratings associated with behavioral listening effort, as indicated by a significant correlation (Table 3) and by the same pattern of results as the response time data (see Figure 2D, bottom right panel compared to Figure 1B, right panel). Interestingly, the direction of the relationship between behavioral effort and subjective ratings of time to complete the task was unexpected. In adults, a decrease in perceived time is associated with higher task demands (Block et al., 2010; Sucala et al., 2011). Thus, it would be expected ratings of time would be negatively associated with response times during the dual-task paradigm; ratings of time would *increase* when listening effort *decreased.* The unexpected direction of the relationship might be related to the participant ages in the current study. Previous results demonstrate there are developmental effects of time perception; younger children are less sensitive to the effects of time (Zélanti and Droit-Volet, 2011). Thus, future work is warranted to investigate the interaction between the association between ratings of time, behavioral listening effort, and participant age.

### Self-Reported Listening-Related Fatigue

The third purpose of this study was to evaluate the effects of reverberation on self-reported, listening fatigue. Results revealed increased self-reported listening fatigue with the fatigue question that addressed feeling tired and with a mean score reflecting responses to all five questions. The current data demonstrate that a relatively short, sustained listening task (approximately 25 min) can induce feelings of mental fatigue in both low and moderate reverberant conditions. Participants rated their tiredness as 0.58 points higher, or a 56% increase relative to pre-testing, after a relatively short, sustained listening activity. However, the effect was the same in both environments, consistent with the listening effort data and with the findings of McGarrigle et al. (2017).

The results of this study also demonstrate that the five questions in the Right Now Fatigue Scale described by Bess and Hornsby (2014) are not equally sensitive to the effects of sustained listening. The only question that was sensitive to pre/post-test differences was the one related to tiredness. These data suggest that additional work is needed to validate a “right now” fatigue scale that is appropriate for use with children.

## Conclusion

In summary, the findings of the current study have three important implications. First, in the modest range of SNRs and reverberation times evaluated, the current data do not support the conclusion that increased reverberation results in increased listening effort or fatigue. Instead, only the addition of background noise increased listening effort. These findings suggest the need for future careful investigation into the acoustic limits across which these findings hold true (e.g., longer reverberation times, larger rooms, greater speaker to listener distances). These data, coupled with emerging reports, question the assertion that moderate reverberation is a significant factor related to increases in listening effort. If confirmed, an update to the existing frameworks for understanding listening effort might be warranted. Second, the study results demonstrate that school-aged children’s ratings of perceived performance are similar to their actual performance in controlled laboratory conditions. Moreover, their ratings of “ease of listening” are also related to their word recognition performance. Participants’ perceived test time was the best candidate for a proxy of behavioral listening effort, but more work is necessary to evaluate the validity and reliability of the question. Finally, a relatively brief, focused listening task can induce listening-related fatigue, as indicated by subjective ratings of “tiredness” and an overall right now fatigue score. In total, these data offer no evidence that increasing reverberation to moderate levels increases listening effort or fatigue, but the data do support the reduction of background noise in classrooms.

## Data Availability

The raw data supporting the conclusions of this manuscript will be made available by the authors, without undue reservation, to any qualified researcher.

## Ethics Statement

This study was carried out in accordance with the recommendations of the Behavioral Health Sciences Committee of the Vanderbilt University Medical Center’s Institutional Review Board with written informed consent from all participants’ parents/guardians and written informed assent of all participants. The informed consent and assent were in accordance with the Declaration of Helsinki. The protocol was approved by the Behavioral Health Sciences Committee of Vanderbilt University Medical Center’s Institutional Review Board.

## Author Contributions

EP, BB, BH, and TR designed the study. BB was primarily responsible for the data collection. EP analyzed the data. EP, BB, SM, TR, and BH wrote and edited the manuscript.

## Conflict of Interest Statement

The authors declare that the research was conducted in the absence of any commercial or financial relationships that could be construed as a potential conflict of interest.
